# Latrine Utilization and Associated Factors in Mehal Meda Town in North Shewa Zone, Amhara Region, Ethiopia, 2019

**DOI:** 10.1155/2020/7310925

**Published:** 2020-06-18

**Authors:** Ayele Mamo Abebe, Mesfin Wudu Kassaw, Abinet Dagnew Mekuria, Sisay Shewasinad Yehualshet, Endegena Abebe Fenta

**Affiliations:** ^1^Primary author: Department of pediatrics nursing, Debre Berhan university, Amhara, Ethiopia; ^2^Nursing department, Woldia University, Amhara region, Ethiopia; ^3^Public health department, Debre Berhan university, Amhara Region, Ethiopia; ^4^Department of pediatrics nursing, Debre Berhan university, Amhara, Ethiopia; ^5^Debre Birhan health science college, Debre Birhan, Amhara region, Ethiopia

## Abstract

**Background:**

Worldwide lack of sanitation is a serious health risk, affecting billions of people around the world, particularly the poor and disadvantaged of people around the world. In Sub-Saharan Africa, the number of people who defecate remains the open field 215 million. According to the 2016 Ethiopian Demographic and Health Surveys report, 56% of the rural households use unimproved toilet facilities. One in every three households in the country has no toilet facility. However, achieving real gains in increasing latrine use and quality remained as a challenge. This study was used to assess the latrine utilization and associated factors in Mehal Meda town in North Shewa zone, Amhara region, Ethiopia, 2019.

**Result:**

In this study, a total of 558 participants were included. Out of households, 509 (91.2%) utilized their latrine facility. On the other way, 503 (98.8%) households utilized latrine regularly. Significant variables that were associated to latrine utilization were the occupational status of head of households, observing feces around the compound/latrine, duration of latrine utilization, shape and structure of latrine facility, latrine status during observation, and distance between water well and latrine. According to this study, the magnitude of latrine utilization in Mehal Meda district was 91.2%. It was lower than Ethiopia national expected target of MDGs (100%). Significant variables that were associated to latrine utilization were occupational status of head of households, observing feces around the compound/latrine, shape and structure of latrine facility, latrine status during observation, and distance between water well and latrine facility. Therefore, health education about latrine utilization and its advantage should be given for community in the study area.

## 1. Background

Latrine utilization is defined as the use of the latrine by all the family members in the households [1]. Approximately, 1.1 billion people did not use any facility at all and practiced open-defecation [1, 2]. Globally, about 2.3 billion people who still have no basic sanitation service either practice open defecation (892 million) [3–5]. Moreover, billions of people have continued their life without the basic sanitation services in the world [6–8].

In Sub-Saharan Africa (SAA) like Ethiopia, 76% of the rural population did not use a better-quality hygiene facility, and people were exposed for diarrheal diseases in high burden especially under five children [9–12]. The majority of households, 91% rural and 54% urban, use nonimproved latrine facilities [13, 14]. Based on other studies, the number of people practicing open defecation in southern Asia has declined moderately from 1990, but in Sub-Saharan Africa, the number of people practicing open defecate increased from then in 1990 (increased by 26%) [15, 16].

In Ethiopia, there was progress in reducing child mortality from 123 deaths of less than five years of children per 1,000 live births in 2005 [17]. In other rural studies, 56% of the rural households in Ethiopia use unimproved toilet facilities [18–20]. The recent data Mini EDHS indicates that, in Ethiopia, more than half 55% of households (56.7% in rural and 4.4% in urban areas) access to unimproved sanitation [21]. The government of Ethiopia had set to achieve a national target of 100 percent sanitation coverage in both rural and urban areas and made different effort to achieve it by 2015 [22–24].

As 2011 EDHS finding, the coverage latrine utilization in SNNP, Amhara, Tigray, and Oromia was 56%, 46%, 41%, and 40%, respectively [25]. Similarly, in the study done in Aneded district, the level of latrine utilization was 63% [26]. Also, in the study done in Laelai Maichew Woreda, the age categories ranges from 36 to 50 years had shown significant association to the use of latrine [27].

On the other side, in a study done SNNPRS, participants who had clean latrine facilities were 1.2 fold higher to use than those with unclean once [28] and 1.5 times more likely to have a larger family than nonadopting neighbors [29]. Similarly, a study conducted in Hulet Ejju revealed that 20% of the households have utilized latrine [30]. But there is no previous study in this study area about latrine utilization. Therefore, this study is aimed to assess latrine utilization and associated factors in Mehal Meda town in North Shewa zone, Amhara region, Ethiopia, 2019.

## 2. Methods and Materials

### 2.1. Study Area and Study Period

This cross-sectional study was conducted in Mehal Meda town district is located at 361 km north east of Addis Ababa and about 180 km north of the Debre Birhan town from January 15 to January 30, 2019. There are 4 kebeles in the district. In 2011, the town administration office report total population is about 40394, and the total number of households is 10,069. All households that had latrine facilities in Mehal Meda town were the source of population.

### 2.2. Sample Size

Sample size was calculated using a single population proportion formula. The following assumption was taken to calculate the sample: *P* = 67.4% [31], confidence interval (CI) = 95%, and marginal error (D) = 5%.

(1) $$\begin{array}{c} n=\frac{{\left(Z_{a/2}\right)}^{2}P\left(1-P\right)}{d^{2}}=\frac{{\left(1.96\right)}^{2}\left(0.674\right)\left(0.326\right)}{{\left(0.05\right)}^{2}}=338 \end{array}$$

The sample size was 558 by using 1.5 design effect and adding 10% nonresponse rate.

### 2.3. Sampling Procedure

**Figure 1:**
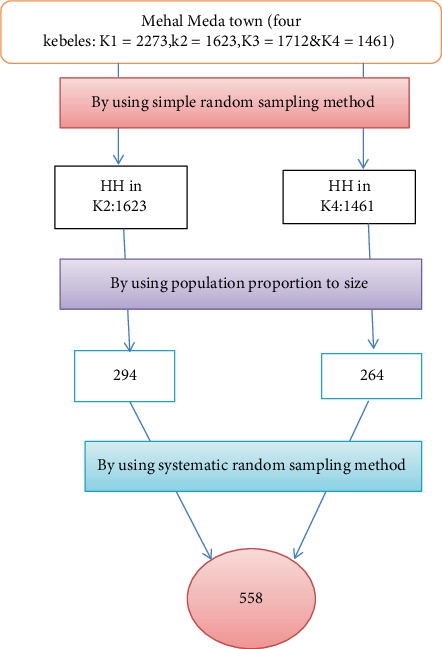
Schematic representation of sampling procedure for assessment of latrine utilization and associated factors in Mehal Meda town in North Shewa zone, Amhara region, Ethiopia, 2019.

The multistage sampling method was employed. Mehal Meda town has 4 kebeles. Then, by using a simple random sampling technique, two kebeles were selected from those kebeles. Households selected using systematic random sampling. The sampling interval (*K*) was gained by dividing each selected Kebele's household number to the sample size, so *k* = *N*/*n* = every 6th household visited until we got 558 Households (Figure 1).

## 3. Schematic Presentation of Sampling Procedure

### 3.1. Data Collection Tool and Procedure

An interview using a structured questionnaire was used by adapted from previous similar literatures [16, 28, 32]. Pretest was done on 5% (*n* = 28) in nonselected kebeles. Data collectors and supervisors had got training for one day on how they collect the data. The principal investigators were strictly following the data collection every day.

### 3.2. Data Quality Assurance

Questionnaire was prepared in English version and translated in to Amharic and back to English to check its consistency. It was checked by senior researchers, and it was pretested on 5% of similar households. The collected data was checked for completeness and finally monitored the overall quality of data collection by the principal investigators.

### 3.3. Data Processing and Analysis

Data were checked for completeness and entered in to SPSS software version 22 for data analysis. Frequency and table used to describe the study population in relation to the relevant variables. Odds ratio with their 95% of CI was computed, and variables having *p* value less than 0.05 in the multiple logistic regression models were considered as significantly associated with the dependent variable.

## 4. Result

### 4.1. Socio-Demographic Characteristics of Respondents

The response rate of this study was 100%, and the majority of participants were found in the age group of 27-35. Mostly, 413 (74%) were males. Regarding of religion, 537 heads of households (96.2%) were Orthodox Christiane, whereas 549 (98.4%) heads of households were Amhara in ethnicity (Table 1).

### 4.2. Socio-Economic Characteristics of Respondents

In this study, 176 (31.5% %) heads of households had diploma and above educational status, and 127 (22.8%) heads of households attended grades 9-12. About the occupational status of heads of households, 287 (51.4%) had private work, whereas 236 (42.3%) were government employees. (Table 2).

### 4.3. Latrine Condition and Feces Disposal Characteristics of Respondents

From the heads of households, 246 (44.1%) utilized latrine below one year. Based on this study, 202 (36.2%) were rectangular metal sheet, and 160 (28.7%) latrines had rectangular hat shape. From the observed households, 472 (84.6%) had no shown feces around the compound. On the other way, 503 (90%) households utilized latrine regularly, and 526 (94.3%) households had no handwashing facility for latrine (within 3 meters). About the cleanness of latrine, 337 (60.4%) latrines were clean (Table 3).

### 4.4. Behavioral and Environmental Factors

The majority of heads of households, 250 (44.8%), claimed to wash their hands after toilet use, whereas 72 (12.9%) heads of households washed their hands during at four critical times. Five hundred thirty-four households (95.7%) lived near to health center with a distance of below 5 km. Similarly, 545 (97.7%) households lived near to the health post with a distance of below 5 km (Table 4).

### 4.5. Factors Associated with Latrine Utilization

In bivariate logistic regression analysis, 14 variables were significantly associated with latrine utilization. However, in multivariable binary logistic regression analysis, educational status of household's head, occupational status of household's head, duration of latrine utilization, cleanness of latrine, latrine status during observation, and distance between water well and latrine facility were significantly associated with latrine utilization with a *p* value <0.05.

Concerning the educational status, the illiterate household heads were 21 [AOR = 20.65, 95% CI: 1.382, 78.479] times more likely to use than those who have diploma and above educational status.

According to this study, household leaders whose work was farmer and government workers were 23 [AOR = 22.651, 95% CI: 2.283, 54.734] and 10 [AOR = 10.305, 95% CI: 2.354, 45.121] times more likely to use latrine than those who have private work, respectively.

Based on this study, the duration of latrine utilizing was 1-3 years were 78.2% less likely to use than those who have 3 years and above duration of use [AOR = 0.218, 95% CI: 0.061, 0.771]. On the other hand, the clean latrines were 9 [AOR = 8.846, 95% CI: 2.919, 26.802] times more likely to use latrine than the counters.

According to this study, households that had good and fair latrine facilities were 25 [AOR = 25.486, 95% CI: 6.268, 103.633] and 14 [AOR = 14.440, 95% CI: 4.233, 49.253] times more likely to utilize latrine than those who had bad latrine facilities. The households that have water well with a distance of below 15 meter from latrine facility were 5 [AOR = 4.469, 95% CI: 1.622, 12.312] times more likely used than the counter (Table 5).

## 5. Discussion

According to this study, the latrine utilization of Mehal Meda town was 91.2%. It was a little bit more than the result of community-based cross-sectional studies in Hulet Ejju Enessie, Aneded district, and in SNNPRS, Southern Ethiopia [26, 28, 30]. The reason could be attributed to the method and areas of the study.

According to this study, five hundred thirty-four households (95.7%) lived near to health center with a distance of below 5 kms. Five hundred thirty-four (95.7%) households had latrine with a distance of below 6 meter. Similarly, in Aneded district study, 55.6% participants lived near to health center with a distance of below 5 km [26]. The possible reason may be due to participants who have enough water sources and who were nearest to the health center/post were used latrine clearly than far from health center/health post/low water source.

Base on this study, 250 (44.8%) heads of households claimed to wash their hands after toilet use, whereas 72 (12.9%) heads of households washed their hands during at four critical times. This finding was lower than the studies done in different parts of Ethiopia [26, 32, 33].

According to this study, the illiterate household heads were 21 [AOR = 20.65, 95% CI: 1.382 78.479] times more likely to use than those who have diploma and above educational status. This result was not supported with the studies done in Aneded district, Laelai Maichew Woreda, and SNNPRS [26–28]. This may be due to the illiterate people may give more attention to use latrine than the educated people.

According to this study, household leaders whose work was farmer and government workers were 23 [AOR = 22.651, 95% CI: 2.283, 54.734)] and 10 [AOR = 10.305, 95% CI: 2.354, 45.121] times more likely to use latrine than those who have private work, respectively. This variable was not shown its association in other studies.

Based on this study, the duration of latrine utilizing was 1-3 years were 78.2% less likely to use than those who have 3 years and above duration of use [AOR = 0.218, 95% CI: 0.061, 0.771]. This variable had not shown its association in other studies.

According to our study, the participants who have clean latrines were 9 [AOR = 8.846, 95% CI: 2.919, 26.802] times more likely to use latrine than the counters. This study was in lined with a study done in SNNPRS [27]. The possible reason may be due to the clean latrine more attractive and comfortable to use than the unclean toilets.

According to this study, households that had good and fair latrine facilities were 25 [AOR = 25.486, 95% CI: 6.268, 103.633] and 14 [AOR = 14.440, 95% CI: 4.233, 49.253] times more likely to utilize latrine than those who had bad latrine facilities. This study was supported by the study done in Aneded district in Ethiopia [26]. This may be due to the reason that the good and fair latrine was attractive and clean to use by families than the counters.

This study revealed that the households that have water well with a distance of below 15 meters from latrine facility were 5 [AOR = 4.469, 95% CI: 1.622, 12.312] times more likely used than the counter. This variable was not shown its association in other studies. The possible reason may be due to participants who have enough water sources.

## 6. Conclusion

Based on this study, the latrine utilization of Mehal Meda district was 91.2%. It was lower than Ethiopia national expected target of MDGs (100%). Occupational status of head of households, observing feces around the compound/latrine, duration of latrine utilization, shape and structure of latrine facility, latrine status during observation, and distance between water well and latrine facility had a significant association with latrine utilization. Therefore, health education should be given on associated findings to get full coverage latrine utilization in this woreda.

### 6.1. Limitation of the Study

The study design is cross-sectional. So, it has its drawback (this does not show which one was come first effect or cause).

## Figures and Tables

**Table 1:** Socio-demographic characteristics of respondents in Mehal Meda town, North Shewa, Ethiopia, 2019 (*N* = 558).

Variables	Category	Frequency	Percent
Age	18-26	41	7.3
	27-35	157	28.1
	36-44	131	23.5
	45-53	147	26.3
	=>54	82	14.7

Sex	Male	413	74.0
	Female	145	26.0

Marital status	Never married	45	8.1
	Married	386	69.2
	Divorced/separated	86	15.4
	Widowed	41	7.3

Religion	Orthodox	537	96.2
	Protestant	21	3.8

Ethnicity	Amhara	549	98.4
	Oromo	9	1.6

Age of family members	Age of males >=5 yrs.	118	21.1
	Age of females >=5 yrs	147	26.3
	Both males and females age >=5 yrs	239	42.8
	Age of 2-5 years children	54	9.7

**Table 2:** Socio-economic characteristics of respondents in Mehal Meda town, North Shewa, Ethiopia, 2019 (*N* = 558).

Variables	Category	Frequency	Percent
Educational status of head household	Illiterate	32	5.7
	Can read and write	118	21.1
	Grades 1-8	105	18.8
	Grades 9-12	127	22.8
	Diploma and above	176	31.5

Occupation of head household	Farmer	35	6.3
	Government employee	236	42.3
	Private	287	51.4

Family monthly income	=<2000	236	42.3
	2001-3500	124	22.2
	=>3501	198	35.5

Family size	=<3	230	41.2
	=>4	328	58.8

Presence of under five children in households	Yes	183	32.8
	No	375	67.2

**Table 3:** Latrine condition and feces disposal characteristics of respondent in Mehal Meda town, North Shewa, Amhara, Ethiopia, 2019.

Variables	Category	Frequency	Percent
Duration of using latrine	Below 1 year	246	44.1
	1-3 years	88	15.8
	Above 3 years	224	40.1

Shape and structure of latrine facility	Traditional hat	120	21.5
	Rectangular hat	160	28.7
	Rectangular metal sheet	202	36.2
	Irregular structure and shape	76	13.6

Observation of any feces around the compound/latrine	Yes	86	15.4
	No	472	84.6

Observation of uncovered foot-path to latrine	Yes	11	2.0
	No	547	98.02

Observation of latrine status	Good	189	33.9
	Fair	214	38.4
	Bad	155	27.8

Status of latrine utilization	Utilized	509	91.2
	Not utilized	49	8.8

Frequency of latrine usage(*N* = 509)	Regularly used	503	90
	Irregularly used	6	1.2

Type of latrine	Flush/pour flush to septic tank/sewer line	28	5.0
	Traditional pit latrine with cemented slab or stone slab	427	76.5
	Traditional pit latrine with wood log and earth cover	92	16.5
	Composting	11	2.0

Availability of hand washing facility for latrine (within 3 meters)	Yes	32	5.7
	No	526	94.3

A vent pipe for the latrine	Yes	23	4.1
	No	535	95.9

Cleanliness of latrine facility	Yes	337	60.4
	No	221	39.6

Arrangement of the latrine	Private latrine/inside the living house	23	4.1
	Private latrine/outside the living house	470	84.2
	Shared with other households/communal	27	4.8
	Shared with the public	38	6.8

Latrine affected by natural disaster	Yes	32	5.7
	No	526	94.3

Latrine accessible to all	Yes	509	91.2
	No	49	8.8

Splash of urine or water on the latrine slab/floor	Yes	335	60.0
	No	223	40.0

**Table 4:** Behavioral and environmental factors of respondents in Mehal Meda town North Shewa, Amhara, Ethiopia, 2019 (*N* = 558).

Variables	Category	Frequency	Percent
Hand washing time	After toilet use	250	44.8
	After care of the child	121	21.7
	Before food making and before child feeding	115	20.6
	During at four critical time	72	12.9

Distance between health center and village (households)	Below 5 km	534	95.7
	Between 5-20 km	24	4.3

Distance between health post and village (households)	Below 5 km	545	97.7
	Between 5-20 km	13	2.3

Distance between latrine and the house	Below 6 m	534	95.7
	Between 6 and 12 m	24	4.3

Having water well in household	Yes	208	37.3
	No	350	62.7

Distance between water well and latrine facility	Below 15 m	289	51.8
	Between 15 and 20 m	269	48.2

**Table 5:** Factors associated with latrine utilization in Mehal Meda town, North Shewa, Amhara, Ethiopia, 2019 (*N* = 558).

Variables	Latrine utilization					
	Yes	Not	*p* value	COR (95% CI)	*p* value	AOR (95% CI)
Marital status						
Never married	43	2		1		1
Married	357	29	0.456	0.573 (0.132, 2.484)	0.254	0.300 (0.038,2.378)
Divorced	70	16	0.040	0.203 (0.045, 0.929)	0.138	0.188 (0.021, 1.709)
Widowed	39	2	0.924	0.907 (0.122, 6.751)	0.884	1.257 (0.059, 26.769)
Educational status of household's head						
Illiterate	31	1	0.632	1.671 (0.204, 13.66)	.028	20.65 (1.382, 78.479)
Can read and write	104	14	0.04	0.40 (0.167, 0.958)	.390	1.937 (0.429,8.748)
Grades 1-8	95	10	0.161	0.512 (0.201, 1.304)	.231	2.808 (0.519, 15.183)
Grades 9-12	112	15	0.038	0.402 (0.170, 0.951)	.508	1.728 (0.342, 8.722)
Diploma and above	167	9		1		1
Occupation of household's head						
Farmer	34	1	0.124	4.88 (0.648, 36.725)	0.008	22.651 (2.283, 54.734)
Government employee	224	12	0.004	2.677 (1.359, 5.273)	0.002	10.305 (2.354, 45.121)
Private	251	36		1		1
Types of latrine						
Flush/pour flush to septic tank/sewer line	4	24	0.349	2.250 (0.412, 12.284)	0.947	1.142 (0.023, 55.699)
Traditional pit latrine with cemented slab or stone slab	24	403	0.009	6.297 (1.569, 25.264)	0.253	6.758 (0.255, 179.049)
Traditional pit latrine with wood log and earth cover	18	74	0.551	1.542 (0.371, 6.400)	0.556	2.684 (0.100, 72.135)
Composting	3	8		1		1
Arrangement of latrine						
Private latrine/inside the living house	1	22	0.043	8.963 (1.073, 74.904)	0.067	12.135 (0.841, 175.009)
Private latrine/outside the living house	30	440	0.001	5.975 (2.705, 13.201)	0.085	3.267 (0850, 12.550)
Shared with other households/communal	7	20	0.789	1.164 (1.164, 0.384, 3.532)	0.500	0.531 (0.085, 3.33)
Shared with the public	11	27		1		1
Duration of latrine utilizing						
Below 1 year	236	10	0.001	3.371 (1.598, 7.112)	0.162	2.265 (0.721, 7.121)
1-3 years	77	11	0.001	1.000 (0.474, 2.108)	0.018	0.218 (0.061, 0.771)
Above 3 years	196	28		1		1
Cleanness of latrine						
Yes	10	327	0.001	7.07 (3.418, 14.367)	.001	8.846 (2.919, 26.802)
No	39	182		1		1
Splash of urine or water around the slab/latrine floor						
Yes	37	298		1		1
No	12	211	0.023	2.183 (1.112, 4.286)	0197	0.481 (0.159, 1.462)
Latrine affected by natural disaster						
Yes	7	25		1		1
No	42	484	0.01	3.227 (1.318, 7.900)	0.819	0.815 (0.141, 4.714)
Observing feces around the latrine						
Yes	59	27	0.001	0.107 (0.057, 0.200)	0.797	0857 (0.266, 2.767)
No	450	22		1		1
Shape and structure of latrine facility						
Traditional hat	108	12	0.496	1.364 (0.558, 3.332)	0.223	2.378 (0.590, 9.581)
Rectangular hat	143	17	0.569	1.275 (0.554, 2.934)	0.741	1.261 (0.318, 4.992)
Rectangular metal sheet	192	10	0.023	2.909 (1.159, 7.300)	0.335	1.926 (0.508, 7.325)
Irregular structure and shape	66	10		1		1
Latrine status during observation						
Good	184	5	0.001	12.372 (4.74, 32.29)	0.001	25.486 (6.268, 103.633)
Fair	209	5	0.001	14.053 (5.39,36.64)	0.001	14.440 (4.233, 49.253)
Bad	116	39		1		1
Distance between water well and latrine facility						
Below 15 m	278	11	0.001	4.157 (2.078, 8.317)	0.004	4.469 (1.622, 12.312)
B/n 15 and 20 m	231	38		1		1

## Data Availability

All data are accessed in this manuscript.

## Abbreviations

AOR: Adjusted odds ratio
CI: Confidence interval
DALYs: Disability –adjusted life years
EDHS: Ethiopian demographic health survey
MDG's: Millennium development goal
OD: Open defecation
OR: Odds ratio
SNNP: Southern nations, nationalities, and people
SPSS: Statistical package for social science
SSA: Sub Sahara Africa
UNICEF: United nation international Children's emergency fund
WASH: Water, sanitation and hygiene
WHO: World Health Organization.
